# Calcium Dyshomeostasis and Lysosomal Ca^2+^ Dysfunction in Amyotrophic Lateral Sclerosis

**DOI:** 10.3390/cells8101216

**Published:** 2019-10-08

**Authors:** Valentina Tedeschi, Tiziana Petrozziello, Agnese Secondo

**Affiliations:** Division of Pharmacology, Department of Neuroscience, Reproductive and Odontostomatological Sciences, School of Medicine, “Federico II” University of Naples, Via S.Pansini 5, 80131 Napoli, Italy; valentinatedeschi5891@gmail.com (V.T.); tiziana.petrozziello@unina.it (T.P.)

**Keywords:** Ca^2+^ homeostasis, lysosomes, endoplasmic reticulum (ER), Ca^2+^-storing organelles, amyotrophic lateral sclerosis (ALS)

## Abstract

Recent findings in the understanding of amyotrophic lateral sclerosis (ALS) revealed that alteration in calcium (Ca^2+^) homeostasis may largely contribute to motor neuron demise. A large part of these alterations is due to dysfunctional Ca^2+^-storing organelles, including the endoplasmic reticulum (ER) and mitochondria. Very recently, lysosomal Ca^2+^ dysfunction has emerged as an important pathological change leading to neuronal loss in ALS. Remarkably, the Ca^2+^-storing organelles are interacting with each other at specialized domains controlling mitochondrial dynamics, ER/lysosomal function, and autophagy. This occurs as a result of interaction between specific ionic channels and Ca^2+^-dependent proteins located in each structure. Therefore, the dysregulation of these ionic mechanisms could be considered as a key element in the neurodegenerative process. This review will focus on the possible role of lysosomal Ca^2+^ dysfunction in the pathogenesis of several neurodegenerative diseases, including ALS and shed light on the possibility that specific lysosomal Ca^2+^ channels might represent new promising targets for preventing or at least delaying neurodegeneration in ALS.

## 1. Introduction

Amyotrophic lateral sclerosis (ALS) is a non-cell autonomous and multisystem disease with an unclear triggering factor. Several pathophysiological causes contributing to the progression of the disease have been identified, including genetic mutations [1], excitotoxicity [2], oxidative stress [3], deregulated immune/inflammatory processes [4], and mitochondrial dysfunction [5]. Interestingly, most of these processes interfere with calcium (Ca^2+^) homeostasis. Accordingly, increased activity of P/Q-type voltage-gated Ca^2+^ channels [6], reduced number of excitatory amino acid transporters [7], specific alterations in the subunit composition of the α-amino-3-hydroxy-5-methyl-4-isoxazole propionic acid (AMPA) glutamate receptor that make it permeable to Ca^2+^, and increased glutamate level may lead to an excessive Ca^2+^ influx in ALS [8]. Importantly, dysfunctional Ca^2+^ level may induce abnormal misfolding of proteins in ALS, thus facilitating their toxic aggregation [9]. Another aspect that deserves attention is that an increased cytosolic Ca^2+^ level may impair the buffer systems of the endoplasmic reticulum (ER) further elevating the intracellular concentration of the ion [10] and causing ER Ca^2+^ leak. On the other hand, nanomolar concentration of Ca^2+^ released from ER is able to trigger the prosurvival signaling pathway at motor neuronal level in an experimental model of ALS [11]. Therefore, global calcium dyshomeostasis linked to organellar dysfunction, being one of the main processes present in several forms of ALS, could be actually considered the unique link among the pathogenetic mechanisms so far known. Indeed, stabilization of such ionic balance might be a promising possibility in ALS therapy. Very recently, lysosomal Ca^2+^ dysfunction has emerged as an important pathological change leading to neuronal demise in ALS [12], highlighting the role of this tiny organelle in the maintenance of global cellular homeostasis. The idea of lysosomes as mere degradative structures has dramatically changed and they are now being considered as dynamic organelles deputed to Ca^2+^ homeostasis and several Ca^2+^-dependent functions [13]. Therefore, it is not surprising that lysosomal dysfunction underlies various organic diseases. For instance, lysosomal Ca^2+^ dyshomeostasis may be considered the principal cause of autophagy deregulation, mainly involved in several neurodegenerative disorders. This review will focus on some new findings linking Ca^2+^ dyshomeostasis and lysosomal dysfunction to downstream mechanisms leading to neurodegeneration in ALS. Here, lysosomal Ca^2+^ homeostasis and its machinery will be presented as new therapeutic targets potentially useful in ALS.

## 2. Lysosomal Ca^2+^ within the Global Ca^2+^ Homeostasis: Interplay and Function

Ca^2+^ is the unique metal ion having a very huge concentration gradient across the plasma membrane of virtually all cells [14]. Maintenance of this ionic gradient is an energy-dependent phenomenon that requires controlled intracellular calcium signaling machinery [15]. This is assured by specific ionic mechanisms located on the plasma membrane and specialized organelles [13].

Furthermore, transient increases in the intracellular Ca^2+^ concentration ([Ca^2+^]_i_) are used as specific signals regulating various biological outputs such as metabolic activity, cell growth and differentiation, transmitter release, and long-term modification of synaptic efficiency. On the other hand, alteration in [Ca^2+^]_i_ is even implicated in neuronal damaging occurring in the neurodegenerative process.

In excitable cells such as neurons and cardiomyocytes, these Ca^2+^ signals are initiated by the occurrence of action potentials opening voltage-dependent Ca^2+^ channels on plasma membrane at the level of specialized domains. Moreover, [Ca^2+^]_i_ increase also depends on release from intracellular Ca^2+^-storing organelles through specific ion channels, pumps, and exchangers. A further level of complexity is due to an integrated cellular network among these intracellular Ca^2+^-storing organelles. Interestingly, they interact with each other on various cellular tasks through specialized membrane contact sites rather than acting as isolated entities [16,17]. In this respect, a physical and functional interaction between lysosomes and ER has been demonstrated in different cells and tissues [18]. It has been proposed that the close apposition between the two compartments could generate relevant global Ca^2+^ signals within the cell [19]. For instance, disruption of acidic stores function causes a reduction in the calcium transient in cardiomyocytes, resulting in significant blunting of responses to isoprenaline in terms of inotropic effect [20]. Indeed, within the cell, a trigger zone could exist in which acidic calcium stores are tightly packed in an appropriate domain near the ryanodine receptors [21,22,23].

Lysosomes, tiny acidic organelles traditionally studied for their catabolic activity [24], have recently emerged as important Ca^2+^-storing compartments that participate in the regulation of global intracellular Ca^2+^ homeostasis under both physiological and pathological conditions [19,25]. Accordingly, a rapid osmotic permeabilization of lysosomes evokes prolonged and spatiotemporally complex Ca^2+^ signals requiring the intervention of different intracellular receptors [18]. Indeed, the average free Ca^2+^ concentration within their lumen is approximately 500 μM [26], which is comparable to the Ca^2+^ concentration reached in the ER [27].

Lysosomal Ca^2+^ could be released by intracellular cues, including nicotinic acid adenine dinucleotide phosphate (NAADP) [28] and lysosome-enriched phosphoinositide PI(3,5)P_2_, mainly involved in membrane trafficking [29]. This local Ca^2+^ release is crucial for lysosome function, but may be also involved in global Ca^2+^ signaling by interacting with ER Ca^2+^ signaling [13,18,30,31]. This evidence has reinforced the study on the functional relationship between the two organelles in handling Ca^2+^ ions. Very recently, the ER Ca^2+^-sensor STIM1 (stromal-interacting molecule 1) has been identified as a fine regulation mechanism of lysosome/ER coupling [12].

**Figure 1:**
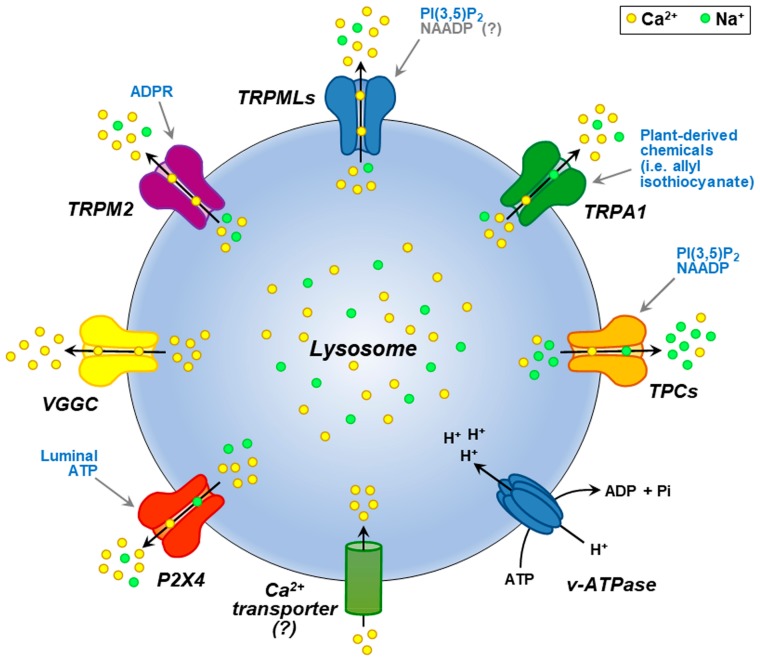
Representation of lysosomal channels and pumps transporting Ca^2+^ and Na^+^ ions. Endogenous agonists are reported in light blue for each of them; hypothetical endogenous agonists are reported in grey. Lysosomal proton-pump V-type ATPase (*v-ATPase*) pumping protons into the lumen is also depicted.

Different Ca^2+^-release channels are localized to the lysosomal membrane (see Figure 1), including the ATP-gated cation channel, P2X4; the member of the melastatin subfamily of TRP (transient receptor potential) ion channels, TRPM2; P/Q-type voltage-gated Ca^2+^ channels (VGCCs); and the non-selective cation channel belonging to the ankyrin subfamily of TRPs, TRPA1. 

Unfortunately, for some of these proteins, direct measurement of their activity is still lacking. The most studied among them is the transient receptor potential mucolipin subfamily 1 (TRPML1) that belongs to the mucolipin subgroup of TRP ion channel family [32]. Moreover, TRPML1 represents the main Ca^2+^-releasing channel used as lysosomal receptor by PI(3,5)P_2_. Interestingly PI(3,5)P_2_ levels are significantly impaired in some ALS forms [33,34]. 

Loss-of-function mutations in TRPML1 cause mucolipidosis type IV (MLIV), a devastating lysosomal storage disorder characterized by neurodegeneration [35]. TRPML1 is widely expressed and predominantly localized on late endosomes and lysosomes, while the other two mammalian members of the mucolipin subfamily, TRPML2 and TRPML3, are also expressed on recycling and early endosomes, respectively [36]. Despite being a non-selective cation channel, TRPML1 displays a high permeability to Ca^2+^ and participates in the regulation of several Ca^2+^-dependent functions within the cell [37]. Therefore, TRPML1-mediated lysosomal Ca^2+^ release may fulfill lysosomal exocytosis [38,39], membrane repair [40], autophagy [41], nutrient sensing [42], oxidative stress sensing [43], lysosome motility, and lysosome tubulation and reformation [44]. Another important type of lysosomal channel is represented by the two-pore channels (TPCs) containing two tandem six-transmembrane domains. Whereas TPC1 is mainly expressed in endosomal compartments, TPC2 is predominantly present on lysosomal membranes [28] and is proposed to be the long-sought NAADP receptor that mediates NAADP-induced Ca^2+^ release from lysosomes [28,45,46,47,48]. Recently, Aston et al. showed the co-localization between NAADP and TPC2 on acidic stores in cardiac ventricular myocytes using live cell microscopy [23]. However, the Ca^2+^ permeability of TPC2 has been questioned by recent studies, thus considering TPC2 as Na^+^-selective channel with a low Ca^2+^ permeability [49,50]. In this respect, mutagenesis studies identified specific molecular determinants for Na^+^ selectivity in TPC2 structure [51].

Once released, Ca^2+^ must be refilled to ensure all lysosomal functions. Recently Atakpa et al. [52] proposed that microdomains of high local Ca^2+^ concentration presented to lysosomes by the ER IP_3_ (Inositol 1,4,5-trisphosphate) receptor (IP_3_R) may facilitate lysosomal Ca^2+^ uptake. However, how lysosomal Ca^2+^ is taken up after its release remains to be answered. Two different refilling mechanisms have been proposed: (a) a putative Ca^2+^–H^+^ exchanger transporting Ca^2+^ ions into lysosomal lumen in an H^+^-dependent manner [25] (see Figure 1) and (b) an unidentified Ca^2+^ transporter at the level of ER-lysosome membrane contact sites activated by ER Ca^2+^ release [53]. This suggests that the functional coupling with the ER is vital for lysosomal function and vice versa. Importantly, an essential role is occupied by ER in nervous system functions and during neurodegeneration [54]. In this respect, an abundance of Ca^2+^ within the ER is essential for nascent protein folding and cell survival [55]. Recently, we have shown that ER plays a crucial role in triggering the Ca^2+^/Akt/ERK1/2 neuroprotective pathway in a model of ALS/Parkinson-dementia complex (ALS/PDC) [11].

Besides the Ca^2+^-storing properties, other functions are controlled by ER in motor neurons. During neuronal circuit development, the spatial localization of ER controls the growth cone cytoskeleton to direct motility of motor neurons [56]. This is due to the intervention of the only known ER calcium sensor STIM1 that, in this circumstance, was acting independently from store-operated calcium entry. Therefore, this finding suggests multiple and unexplored functions for STIM1. Very recently, we have shown that in NSC-34 cells and rat primary motor neurons, TRPML1 channel co-localizes with STIM1 [12], thus suggesting its regulatory function on lysosomal channel activity. Additionally, the specific and irreversible SERCA (sarco-endoplasmic reticulum Ca^2+^ ATPase) inhibitor thapsigargin, by depleting the ER Ca^2+^ store, influences lysosomal Ca^2+^ content [12], thus highlighting the relevance of ER and lysosome connection. This is corroborated by the evidence that dysfunctional Ca^2+^ homeostasis in one of these organelles has a dramatic repercussion on the other store [12]. In fact, there is a persuasion on the strategic role of ER in promoting lysosomal Ca^2+^ refilling in other cellular systems [53].

Many proteins control organellar interplay and are controlled in turn by store functions. For instance, in addition to mitochondria, the calcium-sensitive GTPase dynamin is also associated with lysosomal membrane [57], where it is regulated by lysosomal Ca^2+^ release [58]. In particular, this modulation seems to occur through TRPML1 Ca^2+^ release [59]. It has been demonstrated that, once activated, dynamin tightly controls ER [60], mitochondria [61] and lysosomal fission [62].

Since lysosome size is dynamically regulated by lysosomal membrane fusion and fission [63], the passage of Ca^2+^ ions between these organelles is essential for lysosomal function and global cellular homeostasis. Interestingly, the functional ER/lysosome interplay is not pH-sensitive, since the v-ATPase inhibitors bafilomycin-A or concanamycin-A did not abolish the refilling, but it depends on IP_3_R activity [18,23,64]. Overall, despite the efforts of several researchers leading to important findings, further studies are required to identify all ER/lysosomal partners involved in handling Ca^2+^ ions and controlling lysosomal-related functions.

## 3. Lysosomal Ca^2+^ Signaling and Autophagy

Autophagy is an evolutionarily conserved cellular process through which cytoplasmic materials such as proteins or even complete organelles are directed to lysosomes for degradation. This process allows the clearance of protein aggregates, long-lived proteins, misfolded proteins, and dysfunctional and damaged organelles, and ensures the recycling of their constituents [65,66]. In mammals, autophagy occurs under basal conditions and is implicated in the maintenance of normal cellular homeostasis [65]. However, the process can be stimulated by several physiological and pathological conditions, including starvation, hypoxia, reactive oxygen species (ROS) production, pathogenic infections, various diseases, or by treatment with pharmacological agents like rapamycin and torin-1. Under these conditions, the activation of autophagy allows cells to overcome the stress, thus promoting cell survival. However, an impairment in the autophagic process can contribute to the pathogenesis of several human diseases, including neurodegenerative disorders, cancer, and infectious diseases [65,66].

In mammalian cells, three major subtypes of autophagy have been described, based on the mechanism by which substrates reach lysosomal lumen: Chaperone-mediated autophagy (CMA), microautophagy, and macroautophagy. Only soluble proteins, but not complete organelles, can be delivered to lysosomes via CMA [67], whereas microautophagy and macroautophagy participate in the degradation of both proteins and organelles [68]. In more detail, CMA is characterized by the translocation of cytosolic proteins with the pentapeptide KFERQ motif into lysosomal lumen directly across lysosomal membrane. In this process, protein import is directly mediated by the lysosomal-associated membrane protein type 2A (LAMP2A) translocation complex [66,67]. Microautophagy is a constitutive and sometimes selective process by which whole portions of the cytoplasm are engulfed by direct invagination of lysosomal membrane into tubulovesicular structures [65,69,70]. Macroautophagy, hereafter referred to as autophagy, is the most extensively studied and the best characterized form of autophagy. During this process, a portion of cytoplasm including soluble materials and organelles is sequestered within a phagophore, a de novo-formed double-membraned structure also named isolation membrane. After elongation, the phagophore closes upon itself to form a discrete double-membrane autophagic vesicle, called autophagosome, which entraps sequestered cytosolic cargoes. Subsequently, via cytoskeleton-dependent motion, autophagosome engages and fuses with lysosome in a Ca^2+^-dependent manner, forming a fusion-hybrid organelle called autolysosome. Within the autolysosome, lysosomal enzymes degrade cargoes and the resulting macromolecules are transported back to cytosol for recycling [66,68,71,72,73,74]. When autophagic process is completed, lysosomes are reformed from the fusion-hybrid organelles through lysosome biogenesis [75].

Several studies have shown that autophagy induction is a Ca^2+^-dependent process, during which a crucial role is played by the lysosomal channel, TRPML1 [76]. Indeed, it has been shown that the overexpression of this channel was able to induce an increase in the autophagic flux, whereas its silencing was responsible for a reduction in the autophagic pathway in HeLa cells [76]. Furthermore, in human fibroblasts derived from MLIV patients, there was an impairment in the autophagic pathway, which induced a significant accumulation of autophagic markers p62 (SQSTM1/p62) and LC3-II (1A/1B light chain 3-phosphatidyl ethanolamine conjugate), as a result of increased autophagosome formation and delayed autophagosome fusion with lysosomes. These alterations led to an inefficient autophagosome degradation [77]. A similar increase in p62 and LC3-II was also observed in neuronal cells derived from *mcoln1^-/-^* mouse, a murine model of MLIV. This finding suggested an accumulation of protein aggregates and a defect in autophagy which could account for the neurodegeneration occurring in MLIV [78]. Autophagic defects have also been described in other animal models of MLIV, including *Drosophila trpml* mutant and *Caenorhabditis elegans cup-5* null mutant. In particular, autophagy disruption, observed in *trpml* mutant flies exhibiting an MLIV-like phenotype, was attributable to a reduced degradation of cargoes following fusion between autophagosomes and lysosomes [79]. Similarly, in *C. elegans*, mutations in *cup-5* were responsible for defects in the autophagic pathway. Indeed, in *cup-5* null mutant worms, a variety of autophagic substrates accumulated in enlarged vacuoles similar to late endosomes and lysosomes, thus indicating a dysfunction in autolysosomes proteolytic degradation [80].

The molecular mechanisms underlying TRPML1-mediated regulation of autophagy have been recently described. In particular, lysosomal Ca^2+^ release through the channel, by activating Ca^2+^/calmodulin-dependent Ser/Thr phosphatase calcineurin, promoted the dephosphorylation and subsequent nuclear translocation of TFEB (transcription factor EB) [76], a lysosomal master gene regulator [76,81]. Once in the nucleus, TFEB drives the expression of several genes controlling lysosomal functions, including those involved in the regulation of autophagic pathway [76,81]. Interestingly, TRPML1 inhibition, by preventing cytoplasm-to-nucleus shuttling of TFEB, hampered autophagic genes transcription, thus determining a block in the process [76].

Furthermore, it has been shown that autophagy can also be activated by lysosomal Ca^2+^ release through TRPML1 in response to oxidative stress conditions. Indeed, following an elevation in ROS production or an increase in exogenous oxidants, TRPML1-mediated Ca^2+^ release promoted calcineurin-dependent TFEB nuclear translocation, thus enhancing autophagy and lysosome biogenesis and mitigating oxidative stress. In fact, the subsequent increase in the autophagic flux facilitated the elimination of damaged mitochondria that produce excessive ROS and restored a balanced redox homeostasis [43]. Therefore, TRPML1 could be considered a ROS sensor with a compensative function during oxidative stress.

## 4. Lysosomal Ca^2+^ Dysfunction in Neurodegeneration 

Defects in lysosomal Ca^2+^ handling and release have been indicated as the main causes of the lysosomal storage disorders (LSDs), of which neurodegeneration constitutes an important component. This is mainly due to defects in the key components of lysosomal Ca^2+^ machinery leading to Ca^2+^ dysregulation. In example, defects in lysosomal Ca^2+^ release play a fundamental role in the pathogenesis of MLIV, an autosomal recessive LSD characterized by severe neurodegeneration and caused by loss-of-function mutations in the gene encoding for TRPML1 [35,82,83]. 

Moreover, mutations in key proteins involved in the synthesis of TRPML1 endogenous agonist PI(3,5)P_2_, such as FIG4, are associated with other neurological disorders [84], including some cases of ALS [33,34]. Furthermore, the role of TRPML1 in neurodegeneration is reinforced by the fact that the lysosomal Ca^2+^ channel can also be activated by ROS [43], whose production is strongly upregulated in several neurodegenerative diseases [85].

Besides its well-established role in the neurodegenerative processes underlying MLIV, TRPML1 function is compromised also in other neurodegenerative diseases, including the LSD Niemann–Pick type C (NPC) disease [86] and HIV-associated dementia [87]. 

In particular, in NPC, the low Ca^2+^ levels within the acidic stores, caused by defective lysosomal Ca^2+^ uptake and the subsequent reduction in lysosomal Ca^2+^ release, led to an impairment in late endosomes/lysosomes fusion and to a defective lipid trafficking [88,89]. The participation of TRPML1 in the pathological process underlying the disease has recently emerged, since the accumulation of sphingomyelin inhibited TRPML1 activity, whereas the pharmacological activation of the channel reverted the lysosomal storage phenotype [86].

Similarly, in cellular models of HIV-dementia, the pharmacological activation of TRPML1 was able to promote the clearance of amyloid-β peptides and sphingomyelin at lysosomal level [87].

Other lysosomal Ca^2+^ channels could be involved in the pathogenesis of some neurological diseases. For instance, the inhibition of the voltage-gated Ca^2+^ channel CACNA1A function at lysosomal but not at plasma membrane level led to lysosomal fusion defects [90]. Mutations in this gene are also responsible for episodic ataxia type 2 (EA2), familial hemiplegic migraine-1 (FHM1), and spinocerebellar ataxia type 6 (SCA6) [91].

On the other hand, P2X4 channel is involved in epilepsy and in some forms of ALS [92], although the role of its lysosomal form is still unclear.

A nascent literature has emerged on the role of lysosomal Ca^2+^ dysfunction also in the most frequent neurodegenerative disorders, including Alzheimer’s disease (AD), Parkinson’s disease (PD), and Huntington’s disease (HD) [93,94,95,96].

Moreover, in a mouse model of familial AD, loss-of-function mutations in presenilins (PSEN^-/-^) significantly altered lysosomal Ca^2+^ storage and release, thus causing an impairment in lysosomal fusion processes [97]. It has been proposed that the dysfunction of lysosomal Ca^2+^ observed in PSEN1^-/-^ cells was dependent on the elevation of lysosomal pH, which caused an abnormal Ca^2+^ efflux from lysosomes via a hyperactivated TRPML1 [93]. Furthermore, TRPML1 was downregulated in the APP/PS1 transgenic mice, while channel overexpression was able to rescue memory impairment by hampering neuronal apoptosis. In vitro experiments showed that TRPML1 overexpression rescued primary neurons from lysosomal Ca^2+^ dysregulation and cell death induced by Aβ_1-42_ exposure [98].

Increasing evidences have demonstrated that deregulated Ca^2+^ signaling and lysosomal dysfunction underlie the neurodegeneration associated with PD, probably involving the lysosomal Ca^2+^ channel TPC2. In particular, mutations in PD-linked genes, GBA1 encoding for the lysosomal enzyme glucocerebrosidase and LRRK2 encoding for a protein whose function is still unknown, are responsible for defects in lysosomal Ca^2+^ homeostasis [99,100]. Indeed, it has been shown that dopaminergic neurons generated from fibroblast-derived iPSC carrying GBA1 mutations contained lower lysosomal Ca^2+^ levels compared to wild-type cells and were characterized by a disrupted endolysosomal morphology with enlarged and clustered lysosomes [99]. Lysosomal Ca^2+^ store content was reduced also in *GBA1*-PD fibroblasts similarly to that reported in Niemann–Pick type C1 diseased fibroblasts [31]. Accordingly, Tsunemi and coauthors [101] showed that patient-derived dopaminergic neurons carrying loss of PARK9 function displayed disruption in lysosomal Ca^2+^ homeostasis and increased cytosolic Ca^2+^ levels, thus promoting toxic α-synuclein accumulation. However, the activation of TRPML1-mediated Ca^2+^ release prevented α-synuclein accumulation [101].

Interestingly, in the neurodegenerative process associated with the above-mentioned diseases, lysosomal Ca^2+^ dysfunction determines an impairment of autophagy [98], mainly via TFEB dysregulation [102]. However, the real contribution of the master regulator of the autophagic process and how it could be finely modulated in AD, PD, and other diseases remains to be established.

## 5. Lysosomal Ca^2+^ Dysfunction and Autophagy Defects in ALS

The impairment of human endolysosomal pathway activity with the consequent defects in its downstream functions is now considered a relevant pathogenetic mechanism in ALS. Importantly, it is likely shared as a disease mechanism by both familial (fALS) and sporadic (sALS) ALS forms [103].

Indeed, almost 7 out of 25 genes related to Mendelian inheritance of ALS are involved in endosomal/vesicular biogenesis, maturation, and trafficking. In virtue of this defective clearing mechanism, the main pathological hallmark and potential triggering mechanism of ALS is represented by the accumulation of misfolded proteins in motor neurons. Ubiquitin-positive inclusions linked to SOD1, transactive response DNA-binding protein 43 kDa (TDP-43), fused in sarcoma (FUS) protein, and C9orf72 have been reported in fALS [104]. However, abnormal accumulation of the wild-type form of some proteins, such as TDP-43 and SOD1, has also been observed in sALS [105,106,107]. In most cases, TDP-43 represents the major component of these inclusions. Interestingly, TDP-43 accumulation per se impairs the endolysosomal pathway, resulting in lysosomal dysfunction and lethal autophagy [108]. In fact, the aggregation of ALS-related genes triggers a hyperactive induction of autophagy suggestive of AMPK activation and mTOR repression in motor neurons of SOD1^G85R^ mice [109] that, in the end, resulted in the engulfment of the autophagic flux just like a wheel engulfed in desert sand. Consistently, upregulation of beclin 1 has been found in spinal cord and brainstem fractions of SOD1^G93A^ animals in which p62 and LC3-II levels were elevated [110]. 

Furthermore, C9orf72 insufficiency negatively modulated lysosomal exocytosis process [111], while TFEB, which is normally regulated in a negative way by mTOR activity, was substantially upregulated [112].

Moreover, exposure of motor neurons to cerebrospinal fluid from sALS patients led to a significant dysfunction of the lysosomal proteins hexosaminidase, sialidase, and aryl sulfatase [113]. The same lysosomal defects were determined by the intrathecal injection of sALS cerebrospinal fluid into rat pups [113]. In particular, the authors documented a downregulation and a significant loss of activity of lysosomal enzymes together with mitochondrial dysfunction. This testifies the possible interplay between mitochondria and lysosomes whose dysfunction may lead to motor neuron degeneration in ALS [113].

In support of the neurobeneficial role of a balanced interplay among Ca^2+^-storing organelles, Vollrath et al. showed that depletion of sigma-1 receptor (SigR1), an ER chaperone located at the mitochondria/ER interface, led to an impairment of calcium mobilization, mitochondrial and ER defects, accumulation of undegraded substrates, and altered endosomal trafficking in NSC-34 motor neurons [114]. Furthermore, a SigR1 mutation has been identified as a possible cause of ALS/FTD (amyotrophic lateral sclerosis/frontotemporal dementia) [115], thus indicating this ER protein as a new potential pharmacological target against autophagy dysfunction and Ca^2+^ dyshomeostasis in ALS [116].

All these findings are indicative of the occurrence of defects in cellular cleansing mechanisms in ALS that, in part, are dependent on Ca^2+^ homeostasis dysfunction. In accordance with this idea, Rusmini and coauthors [117] demonstrated that the natural disaccharide trehalose and its analogs promote Ca^2+^-dependent lysosomal clearance of neurotoxic misfolded proteins in motor neurons in a TFEB-dependent manner.

**Figure 2:**
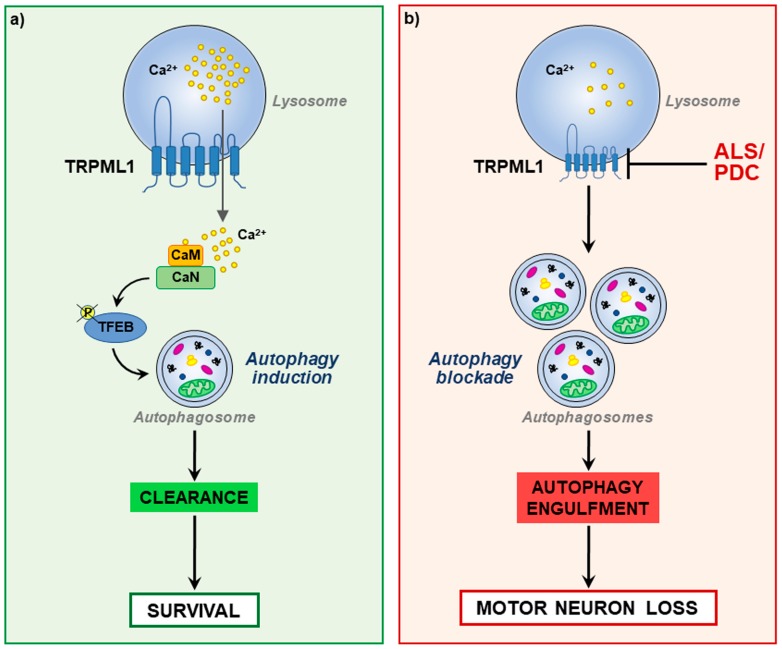
Representation of neuronal TRPML1 (**a**) under physiological conditions in which, after Ca^2+^ release through the channel, a Ca^2+^-dependent activation of TFEB (Transcription factor EB) triggers autophagy thus promoting cell survival; (**b**) in an experimental model of ALS/PDC (Parkinsonism-dementia complex) in which the reduction of TRPML1 expression and activity determines an engulfment of autophagy and motor neuron loss.

Given the relevance of a balanced lysosomal Ca^2+^ homeostasis in the correct modulation of autophagic flux useful in cleansing the cell of defective organelles and misfolded proteins, the involvement of TRPML1 in the pathogenesis of the Guamanian form of ALS (ALS/PDC) has been recently characterized [12]. In this study, dysfunctions in TRPML1 expression and activity have been correlated to the accumulation of autophagic markers in ALS motor neurons as a sign of autophagy engulfment (see Figure 2).

Furthermore, a concomitant defect of ER Ca^2+^ homeostasis occurred in neurons exposed to L-BMAA (the neurotoxin β-methylamino-L-alanine), most likely depending on the disruption of the lysosomal/ER interplay. This suggests that ER store is acting as a major source of lysosomal Ca^2+^ in motor neurons and that dysfunctional Ca^2+^ homeostasis in one of these organelles could have a dramatic repercussion on the other store. Importantly, in this study, early pharmacological stimulation of lysosomal TRPML1, by boosting autophagy, efficiently rescued motor neurons from L-BMAA toxicity and prevented ER stress, a hallmark of several forms of ALS [11,106].

## 6. Concluding Remarks

The impact of endolysosomal system defects on autophagic process is now considered one of the most important pathogenetic mechanisms of several neurological diseases. However, this concept is starting to emerge also for ALS pathogenesis. The exact mechanism underlying lysosomal dysfunction remains to be established. Many studies documented a downregulation of expression and activity of lysosomal enzymes, reduction of lysosomal Ca^2+^ content, and impairment in organellar interplay. In fact, a concomitant dysfunction in ER and mitochondrial Ca^2+^ content has been described together with lysosomal dysfunction. This results in the occurrence of defects in cellular cleansing mechanisms and global Ca^2+^ dyshomeostasis in ALS. In accordance with this evidence, dysfunction of the lysosomal channel TRPML1 is emerging as a pathogenetic mechanism of the disease. Indeed, TRPML1 downregulation determined autophagy engulfment, disruption of lysosomal/ER interplay, and ER stress. These pathognomonic signs are all overcome by boosting autophagy through TRPML1 stimulation. Due to the complexity of the mechanisms involved in lysosomal Ca^2+^ regulation and function, many other efforts are needed to identify new targets within the lysosomal machinery whose modulation could impact Ca^2+^ dyshomeostasis in ALS.

## References

[1] Beleza-Meireles A., Al-Chalabi A. (2009). Genetic studies of amyotrophic lateral sclerosis: Controversies and perspectives. Amyotroph. Lateral Scler..

[2] Choi D.W. (1992). Amyotrophic lateral sclerosis and glutamate-too much of a good thing?. N. Engl. J. Med..

[3] Barber S.C., Shaw P.J. (2010). Oxidative stress in ALS: Key role in motor neuron injury and therapeutic target. Free Radic. Biol. Med..

[4] Hooten K.G., Beers D.R., Zhao W., Appel S.H. (2015). Protective and Toxic Neuroinflammation in Amyotrophic Lateral Sclerosis. Neurotherapeutics.

[5] Carrì M.T., D’Ambrosi N., Cozzolino M. (2017). Pathways to mitochondrial dysfunction in ALS pathogenesis. Biochem. Biophys. Res. Commun..

[6] Li A., Ségui J., Heinemann S.H., Hoshi T. (1998). Oxidation regulates cloned neuronal voltage-dependent Ca2+ channels expressed in Xenopus oocytes. J. Neurosci..

[7] Boillée S., Vande Velde C., Cleveland D.W. (2006). ALS: A disease of motor neurons and their nonneuronal neighbors. Neuron.

[8] Williams T.L., Day N.C., Ince P.G., Kamboj R.K., Shaw P.J. (1997). Calcium-permeable alpha-amino-3-hydroxy-5-methyl-4-isoxazole propionic acid receptors: A molecular determinant of selective vulnerability in amyotrophic lateral sclerosis. Ann. Neurol..

[9] Leal S.S., Cardoso I., Valentine J.S., Gomes C.M. (2013). Calcium ions promote superoxide dismutase 1 (SOD1) aggregation into non-fibrillar amyloid: A link to toxic effects of calcium overload in amyotrophic lateral sclerosis (ALS)?. J. Biol. Chem..

[10] Kiselyov K., Muallem S. (2016). ROS and intracellular ion channels. Cell Calcium.

[11] Petrozziello T., Secondo A., Tedeschi V., Esposito A., Sisalli M., Scorziello A., Di Renzo G., Annunziato L. (2017). ApoSOD1 lacking dismutase activity neuroprotects motor neurons exposed to beta-methylamino-L-alanine through the Ca2+/Akt/ERK1/2 prosurvival pathway. Cell Death Differ..

[12] Tedeschi V., Petrozziello T., Sisalli M.J., Boscia F., Canzoniero L.M.T., Secondo A. (2019). The activation of Mucolipin TRP channel 1 (TRPML1) protects motor neurons from L-BMAA neurotoxicity by promoting autophagic clearance. Sci. Rep..

[13] Raffaello A., Mammucari C., Gherardi G., Rizzuto R. (2016). Calcium at the Center of Cell Signaling: Interplay between Endoplasmic Reticulum, Mitochondria, and Lysosomes. Trends Biochem. Sci..

[14] Jaiswal J.K. (2001). Calcium–How and why?. J. Biosci..

[15] Brini M., Calì T., Ottolini D., Carafoli E. (2014). Neuronal calcium signaling: Function and dysfunction. Cell. Mol. Life Sci..

[16] Schrader M., Godinho L.F., Costello J.L., Islinger M. (2015). The different facets of organelle interplay-an overview of organelle interactions. Front. Cell Dev. Biol..

[17] Han Y., Li M., Qiu F., Zhang M., Zhang Y.H. (2017). Cell-permeable organic fluorescent probes for live-cell long-term super-resolution imaging reveal lysosome-mitochondrion interactions. Nat. Commun..

[18] Kilpatrick B.S., Eden E.R., Schapira A.H., Futter C.E., Patel S. (2013). Direct mobilisation of lysosomal Ca2+ triggers complex Ca2+ signals. J. Cell Sci..

[19] Patel S., Docampo R. (2010). Acidic calcium stores open for business: Expanding the potential for intracellular Ca2+ signaling. Trends Cell Biol..

[20] Capel R.A., Bolton E.L., Lin W.K., Aston D., Wang Y., Liu W., Wang X., Burton R.A., Bloor-Young D., Shade K.T. (2015). Two-pore Channels (TPC2s) and Nicotinic Acid Adenine Dinucleotide Phosphate (NAADP) at Lysosomal-Sarcoplasmic Reticular Junctions Contribute to Acute and Chronic β-Adrenoceptor Signaling in the Heart. J. Biol. Chem..

[21] Kinnear N.P., Boittin F.X., Thomas J.M., Galione A., Evans A.M. (2004). Lysosome-sarcoplasmic reticulum junctions. A trigger zone for calcium signaling by nicotinic acid adenine dinucleotide phosphate and endothelin-1. J. Biol. Chem..

[22] Penny C.J., Kilpatrick B.S., Han J.M., Sneyd J., Patel S. (2014). A computational model of lysosome-ER Ca2+ microdomains. J. Cell Sci..

[23] Aston D., Capel R.A., Ford K.L., Christian H.C., Mirams G.R., Rog-Zielinska E.A., Kohl P., Galione A., Burton R.A., Terrar D.A. (2017). High resolution structural evidence suggests the Sarcoplasmic Reticulum forms microdomains with Acidic Stores (lysosomes) in the heart. Sci. Rep..

[24] De Duve C. (2005). The lysosome turns fifty. Nat. Cell Biol..

[25] Morgan A.J., Platt F.M., Lloyd-Evans E., Galione A. (2011). Molecular mechanisms of endolysosomal Ca2+ signalling in health and disease. Biochem. J..

[26] Christensen K.A., Myers J.T., Swanson J.A. (2002). pH-dependent regulation of lysosomal calcium in macrophages. J. Cell Sci..

[27] Bygrave F.L., Benedetti A. (1996). What is the concentration of calcium ions in the endoplasmic reticulum?. Cell Calcium.

[28] Calcraft P.J., Ruas M., Pan Z., Cheng X., Arredouani A., Hao X., Tang J., Rietdorf K., Teboul L., Chuang K.T. (2009). NAADP mobilizes calcium from acidic organelles through two-pore channels. Nature.

[29] Dong X.P., Shen D., Wang X., Dawson T., Li X., Zhang Q., Cheng X., Zhang Y., Weisman L.S., Delling M. (2010). PI(3,5)P(2) controls membrane trafficking by direct activation of mucolipin Ca(2+) release channels in the endolysosome. Nat. Commun..

[30] Morgan A.J., Davis L.C., Wagner S.K., Lewis A.M., Parrington J., Churchill G.C., Galione A. (2013). Bidirectional Ca^2+^ signaling occurs between the endoplasmic reticulum and acidic organelles. J. Cell Biol..

[31] Kilpatrick B.S., Magalhaes J., Beavan M.S., McNeill A., Gegg M.E., Cleeter M.W., Bloor-Young D., Churchill G.C., Duchen M.R., Schapira A.H. (2016). Endoplasmic reticulum and lysosomal Ca²⁺ stores are remodelled in GBA1-linked Parkinson disease patient fibroblasts. Cell Calcium.

[32] Ramsey I.S., Delling M., Clapham D.E. (2006). An introduction to TRP channels. Annu. Rev. Physiol..

[33] Chow C.Y., Landers J.E., Bergren S.K., Sapp P.C., Grant A.E., Jones J.M., Everett L., Lenk G.M., McKenna-Yasek D.M., Weisman L.S. (2009). Deleterious variants of FIG4, a phosphoinositide phosphatase, in patients with ALS. Am. J. Hum. Genet..

[34] Osmanovic A., Rangnau I., Kosfeld A., Abdulla S., Janssen C., Auber B., Raab P., Preller M., Petri S., Weber R.G. (2017). FIG4 variants in central European patients with amyotrophic lateral sclerosis: A whole-exome and targeted sequencing study. Eur. J. Hum. Genet..

[35] Bargal R., Avidan N., Ben-Asher E., Olender Z., Zeigler M., Frumkin A., Raas-Rothschild A., Glusman G., Lancet D., Bach G. (2000). Identification of the gene causing mucolipidosis type IV. Nat. Genet..

[36] Cheng X., Shen D., Samie M., Xu H. (2010). Mucolipins: Intracellular TRPML1-3 channels. FEBS Lett..

[37] LaPlante J.M., Falardeau J., Sun M., Kanazirska M., Brown E.M., Slaugenhaupt S.A., Vassilev P.M. (2002). Identification and characterization of the single channel function of human mucolipin-1 implicated in mucolipidosis type IV, a disorder affecting the lysosomal pathway. FEBS Lett..

[38] Medina D.L., Fraldi A., Bouche V., Annunziata F., Mansueto G., Spampanato C., Puri C., Pignata A., Martina J.A., Sardiello M. (2011). Transcriptional activation of lysosomal exocytosis promotes cellular clearance. Dev. Cell.

[39] Park S., Ahuja M., Kim M.S., Brailoiu G.C., Jha A., Zeng M., Baydyuk M., Wu L.G., Wassif C.A., Porter F.D. (2016). Fusion of lysosomes with secretory organelles leads to uncontrolled exocytosis in the lysosomal storage disease mucolipidosis type IV. EMBO Rep..

[40] Cheng X., Zhang X., Gao Q., Ali Samie M., Azar M., Tsang W.L., Dong L., Sahoo N., Li X., Zhuo Y. (2014). The intracellular Ca²⁺ channel MCOLN1 is required for sarcolemma repair to prevent muscular dystrophy. Nat. Med..

[41] Medina D.L., Di Paola S., Peluso I., Armani A., De Stefani D., Venditti R., Montefusco S., Scotto-Rosato A., Prezioso C., Forrester A. (2015). Lysosomal calcium signalling regulates autophagy through calcineurin and TFEB. Nat. Cell Biol..

[42] Wang W., Gao Q., Yang M., Zhang X., Yu L., Lawas M., Li X., Bryant-Genevier M., Southall N.T., Marugan J. (2015). Up-regulation of lysosomal TRPML1 channels is essential for lysosomal adaptation to nutrient starvation. Proc. Natl. Acad. Sci. USA.

[43] Zhang X., Cheng X., Yu L., Yang J., Calvo R., Patnaik S., Hu X., Gao Q., Yang M., Lawas M. (2016). MCOLN1 is a ROS sensor in lysosomes that regulates autophagy. Nat. Commun..

[44] Li X., Rydzewski N., Hider A., Zhang X., Yang J., Wang W., Gao Q., Cheng X., Xu H. (2016). A molecular mechanism to regulate lysosome motility for lysosome positioning and tubulation. Nat. Cell Biol..

[45] Brailoiu E., Rahman T., Churamani D., Prole D.L., Brailoiu G.C., Hooper R., Taylor C.W., Patel S. (2010). An NAADP-gated two-pore channel targeted to the plasma membrane uncouples triggering from amplifying Ca2+ signals. J. Biol. Chem..

[46] Zong X., Schieder M., Cuny H., Fenske S., Gruner C., Rötzer K., Griesbeck O., Harz H., Biel M., Wahl-Schott C. (2009). The two-pore channel TPCN2 mediates NAADP-dependent Ca(2+)-release from lysosomal stores. Pflugers Arch..

[47] Lloyd-Evans E., Waller-Evans H., Peterneva K., Platt F.M. (2010). Endolysosomal calcium regulation and disease. Biochem. Soc. Trans..

[48] Penny C.J., Patel S. (2015). Poring over two-pore channel pore mutants. Messenger.

[49] Wang X., Zhang X., Dong X.P., Samie M., Li X., Cheng X., Goschka A., Shen D., Zhou Y., Harlow J. (2012). TPC proteins are phosphoinositide- activated sodium-selective ion channels in endosomes and lysosomes. Cell.

[50] Cang C., Zhou Y., Navarro B., Seo Y.J., Aranda K., Shi L., Battaglia-Hsu S., Nissim I., Clapham D.E., Ren D. (2013). mTOR regulates lysosomal ATP-sensitive two-pore Na(+) channels to adapt to metabolic state. Cell.

[51] Guo J., Zeng W., Jiang Y. (2017). Tuning the ion selectivity of two-pore channels. Proc. Natl. Acad. Sci. USA.

[52] Atakpa P., Thillaiappan N.B., Mataragka S., Prole D.L., Taylor C.W. (2018). IP3 Receptors Preferentially Associate with ER-Lysosome Contact Sites and Selectively Deliver Ca2+ to Lysosomes. Cell Rep..

[53] Garrity A.G., Wang W., Collier C.M., Levey S.A., Gao Q., Xu H. (2016). The endoplasmic reticulum, not the pH gradient, drives calcium refilling of lysosomes. Elife.

[54] Jung J., Michalak M., Agellon L.B. (2017). Endoplasmic Reticulum Malfunction in the Nervous System. Front. Neurosci..

[55] Rashid H.O., Yadav R.K., Kim H.R., Chae H.J. (2015). ER stress: Autophagy induction, inhibition and selection. Autophagy.

[56] Pavez M., Thompson A.C., Arnott H.J., Mitchell C.B., D’Atri I., Don E.K., Chilton J.K., Scott E.K., Lin J.Y., Young K.M. (2019). STIM1 Is Required for Remodeling of the Endoplasmic Reticulum and Microtubule Cytoskeleton in Steering Growth Cones. J. Neurosci..

[57] Itoh K., Adachi Y., Yamada T., Suzuki T.L., Otomo T., McBride H.M., Yoshimori T., Iijima M., Sesaki H. (2018). A brain-enriched Drp1 isoform associates with lysosomes, late endosomes, and the plasma membrane. J. Biol. Chem..

[58] Cousin M.A., Robinson P.J. (2000). Ca(2+) influx inhibits dynamin and arrests synaptic vesicle endocytosis at the active zone. J. Neurosci..

[59] Zou J., Hu B., Arpag S., Yan Q., Hamilton A., Zeng Y.S., Vanoye C.G., Li J. (2015). Reactivation of Lysosomal Ca2+ Efflux Rescues Abnormal Lysosomal Storage in FIG4-Deficient Cells. J. Neurosci..

[60] Yoon Y., Pitts K.R., McNiven M.A. (2001). Mammalian dynamin-like protein DLP1 tubulates membranes. Mol. Biol. Cell.

[61] Cereghetti G.M., Stangherlin A., Martins de Brito O., Chang C.R., Blackstone C., Bernardi P., Scorrano L. (2008). Dephosphorylation by calcineurin regulates translocation of Drp1 to mitochondria. Proc. Natl. Acad. Sci. USA.

[62] Fang X., Zhou J., Liu W., Duan X., Gala U., Sandoval H., Jaiswal M., Tong C. (2016). Dynamin Regulates Autophagy by Modulating Lysosomal Function. J. Genet. Genomics.

[63] Durchfort N., Verhoef S., Vaughn M.B., Shrestha R., Adam D., Kaplan J., Ward D.M. (2012). The enlarged lysosomes in beige j cells result from decreased lysosome fission and not increased lysosome fusion. Traffic.

[64] Kilpatrick B.S., Eden E.R., Hockey L.N., Yates E., Futter C.E., Patel S. (2017). An Endosomal NAADP-Sensitive Two-Pore Ca2+ Channel Regulates ER-Endosome Membrane Contact Sites to Control Growth Factor Signaling. Cell Rep..

[65] Ravikumar B., Sarkar S., Davies J.E., Futter M., Garcia-Arencibia M., Green-Thompson Z.W., Jimenez-Sanchez M., Korolchuk V.I., Lichtenberg M., Luo S. (2010). Regulation of mammalian autophagy in physiology and pathophysiology. Physiol. Rev..

[66] La Rovere R.M., Roest G., Bultynck G., Parys J.B. (2016). Intracellular Ca(2+) signaling and Ca(2+) microdomains in the control of cell survival, apoptosis and autophagy. Cell Calcium.

[67] Cuervo A.M. (2010). Chaperone-mediated autophagy: Selectivity pays off. Trends Endocrinol. Metab..

[68] Yang Z., Klionsky D.J. (2009). An overview of the molecular mechanism of autophagy. Curr. Top. Microbiol. Immunol..

[69] Wong E., Cuervo A.M. (2010). Integration of clearance mechanisms: The proteasome and autophagy. Cold Spring Harb. Perspect. Biol..

[70] Nixon R.A., Yang D.S. (2012). Autophagy and neuronal cell death in neurological disorders. Cold Spring Harb. Perspect. Biol..

[71] Burman C., Ktistakis N.T. (2010). Regulation of autophagy by phosphatidylinositol 3-phosphate. FEBS Lett..

[72] Appelqvist H., Wäster P., Kågedal K., Öllinger K. (2013). The lysosome: From waste bag to potential therapeutic target. J. Mol. Cell Biol..

[73] Carlsson S.R., Simonsen A. (2015). Membrane dynamics in autophagosome biogenesis. J. Cell Sci..

[74] Bootman M.D., Chehab T., Bultynck G., Parys J.B., Rietdorf K. (2018). The regulation of autophagy by calcium signals: Do we have a consensus?. Cell Calcium.

[75] Xu H., Ren D. (2015). Lysosomal physiology. Annu. Rev. Physiol..

[76] Medina D.L., Ballabio A. (2015). Lysosomal calcium regulates autophagy. Autophagy.

[77] Vergarajauregui S., Connelly P.S., Daniels M.P., Puertollano R. (2008). Autophagic dysfunction in mucolipidosis type IV patients. Hum. Mol. Genet..

[78] Curcio-Morelli C., Charles F.A., Micsenyi M.C., Cao Y., Venugopal B., Browning M.F., Dobrenis K., Cotman S.L., Walkley S.U., Slaugenhaupt S.A. (2010). Macroautophagy is defective in mucolipin-1-deficient mouse neurons. Neurobiol. Dis..

[79] Venkatachalam K., Long A.A., Elsaesser R., Nikolaeva D., Broadie K., Montell C. (2008). Motor deficit in a Drosophila model of mucolipidosis type IV due to defective clearance of apoptotic cells. Cell.

[80] Sun T., Wang X., Lu Q., Ren H., Zhang H. (2011). CUP-5, the C. elegans ortholog of the mammalian lysosomal channel protein MLN1/TRPML1, is required for proteolytic degradation in autolysosomes. Autophagy.

[81] Sardiello M., Palmieri M., di Ronza A., Medina D.L., Valenza M., Gennarino V.A., Di Malta C., Donaudy F., Embrione V., Polishchuk R.S. (2009). A gene network regulating lysosomal biogenesis and function. Science.

[82] Bassi M.T., Manzoni M., Monti E., Pizzo M.T., Ballabio A., Borsani G. (2000). Cloning of the gene encoding a novel integral membrane protein, mucolipidin-and identification of the two major founder mutations causing mucolipidosis type IV. Am. J. Hum. Genet..

[83] Sun M., Goldin E., Stahl S., Falardeau J.L., Kennedy J.C., Acierno J.S., Bove C., Kaneski C.R., Nagle J., Bromley M.C. (2000). Mucolipidosis type IV is caused by mutations in a gene encoding a novel transient receptor potential channel. Hum. Mol. Genet..

[84] Jin N., Lang M.J., Weisman L.S. (2016). Phosphatidylinositol 3,5-bisphosphate: Regulation of cellular events in space and time. Biochem. Soc. Trans..

[85] Barnham K.J., Masters C.L., Bush A.I. (2004). Neurodegenerative diseases and oxidative stress. Nat. Rev. Drug Discov..

[86] Shen D., Wang X., Li X., Zhang X., Yao Z., Dibble S., Dong X.P., Yu T., Lieberman A.P., Showalter H.D. (2012). Lipid storage disorders block lysosomal trafficking by inhibiting a TRP channel and lysosomal calcium release. Nat. Commun..

[87] Bae M., Patel N., Xu H., Lee M., Tominaga-Yamanaka K., Nath A., Geiger J., Gorospe M., Mattson M.P., Haughey N.J. (2014). Activation of TRPML1 clears intraneuronal Aβ in preclinical models of HIV infection. J. Neurosci..

[88] Lloyd-Evans E., Morgan A.J., He X., Smith D.A., Elliot-Smith E., Sillence D.J., Churchill G.C., Schuchman E.H., Galione A., Platt F.M. (2008). Niemann-Pick disease type C1 is a sphingosine storage disease that causes deregulation of lysosomal calcium. Nat. Med..

[89] Lloyd-Evans E., Platt F.M. (2011). Lysosomal Ca(2+) homeostasis: Role in pathogenesis of lysosomal storage diseases. Cell Calcium.

[90] Tian X., Gala U., Zhang Y., Shang W., Nagarkar Jaiswal S., di Ronza A., Jaiswal M., Yamamoto S., Sandoval H., Duraine L. (2015). A voltage-gated calcium channel regulates lysosomal fusion with endosomes and autophagosomes and is required for neuronal homeostasis. PLoS Biol..

[91] Rajakulendran S., Kaski D., Hanna M.G. (2012). Neuronal P/Q-type calcium channel dysfunction in inherited disorders of the CNS. Nat. Rev. Neurol..

[92] Sáez-Orellana F., Godoy P.A., Silva-Grecchi T., Barra K.M., Fuentealba J. (2015). Modulation of the neuronal network activity by P2X receptors and their involvement in neurological disorders. Pharmacol. Res..

[93] Lee J.H., McBrayer M.K., Wolfe D.M., Haslett L.J., Kumar A., Sato Y., Lie P.P., Mohan P., Coffey E.E., Kompella U. (2015). Presenilin 1 Maintains Lysosomal Ca(^2+^) Homeostasis via TRPML1 by Regulating vATPase-Mediated Lysosome Acidification. Cell Rep..

[94] Croce K.R., Yamamoto A. (2019). A role for autophagy in Huntington’s disease. Neurobiol. Dis..

[95] Yamada M., Tsuji S., Takahashi H. (2002). Involvement of lysosomes in the pathogenesis of CAG repeat diseases. Ann. Neurol..

[96] Koh J.Y., Kim H.N., Hwang J.J., Kim Y.H., Park S.E. (2019). Lysosomal dysfunction in proteinopathic neurodegenerative disorders: Possible therapeutic roles of cAMP and zinc. Mol. Brain.

[97] Coen K., Flannagan R.S., Baron S., Carraro-Lacroix L.R., Wang D., Vermeire W., Michiels C., Munck S., Baert V., Sugita S. (2012). Lysosomal calcium homeostasis defects, not proton pump defects, cause endo-lysosomal dysfunction in PSEN-deficient cells. J. Cell Biol..

[98] Zhang L., Fang Y., Cheng X., Lian Y., Xu H., Zeng Z., Zhu H. (2017). TRPML1 Participates in the Progression of Alzheimer’s Disease by Regulating the PPARγ/AMPK/Mtor Signalling Pathway. Cell. Physiol. Biochem..

[99] Schöndorf D.C., Aureli M., McAllister F.E., Hindley C.J., Mayer F., Schmid B., Sardi S.P., Valsecchi M., Hoffmann S., Schwarz L.K. (2014). iPSC-derived neurons from GBA1-associated Parkinson’s disease patients show autophagic defects and impaired calcium homeostasis. Nat. Commun..

[100] Gómez-Suaga P., Luzón-Toro B., Churamani D., Zhang L., Bloor-Young D., Patel S., Woodman P.G., Churchill G.C., Hilfiker S. (2012). Leucine-rich repeat kinase 2 regulates autophagy through a calcium-dependent pathway involving NAADP. Hum. Mol. Genet..

[101] Tsunemi T., Perez-Rosello T., Ishiguro Y., Yoroisaka A., Jeon S., Hamada K., Rammonhan M., Wong Y.C., Xie Z., Akamatsu W. (2019). Increased Lysosomal Exocytosis Induced by Lysosomal Ca2+ Channel Agonists Protects Human Dopaminergic Neurons from α-Synuclein Toxicity. J. Neurosci..

[102] Cortes C.J., La Spada A.R. (2019). TFEB dysregulation as a driver of autophagy dysfunction in neurodegenerative disease: Molecular mechanisms, cellular processes, and emerging therapeutic opportunities. Neurobiol. Dis..

[103] Cipolat Mis M.S., Brajkovic S., Frattini E., Di Fonzo A., Corti S. (2016). Autophagy in motor neuron disease: Key pathogenetic mechanisms and therapeutic targets. Mol. Cell. Neurosci..

[104] Peters O.M., Ghasemi M., Brown R.H. (2015). Emerging mechanisms of molecular pathology in ALS. J. Clin. Invest..

[105] Neumann M., Sampathu D.M., Kwong L.K., Truax A.C., Micsenyi M.C., Chou T.T., Bruce J., Schuck T., Grossman M., Clark C.M. (2006). Ubiquitinated TDP-43 in frontotemporal lobar degeneration and amyotrophic lateral sclerosis. Science.

[106] Medinas D.B., Rozas P., Martínez Traub F., Woehlbier U., Brown R.H., Bosco D.A., Hetz C. (2018). Endoplasmic reticulum stress leads to accumulation of wild-type SOD1 aggregates associated with sporadic amyotrophic lateral sclerosis. Proc. Natl. Acad. Sci. USA.

[107] Bosco D.A., Morfini G., Karabacak N.M., Song Y., Gros-Louis F., Pasinelli P., Goolsby H., Fontaine B.A., Lemay N., McKenna-Yasek D. (2010). Wild-type and mutant SOD1 share an aberrant conformation and a common pathogenic pathway in ALS. Nat. Neurosci..

[108] Leibiger C., Deisel J., Aufschnaiter A., Ambros S., Tereshchenko M., Verheijen B.M., Büttner S., Braun R.J. (2018). TDP-43 controls lysosomal pathways thereby determining its own clearance and cytotoxicity. Hum. Mol. Genet..

[109] Hetz C., Thielen P., Matus S., Nassif M., Court F., Kiffin R., Martinez G., Cuervo A.M., Brown R.H., Glimcher L.H. (2009). XBP-1 deficiency in the nervous system protects against amyotrophic lateral sclerosis by increasing autophagy. Genes Dev..

[110] Morimoto N., Nagai M., Ohta Y., Miyazaki K., Kurata T., Morimoto M., Murakami T., Takehisa Y., Ikeda Y., Kamiya T. (2007). Increased autophagy in transgenic mice with a G93A mutant SOD1 gene. Brain Res..

[111] Zhang Y., Burberry A., Wang J.Y., Sandoe J., Ghosh S., Udeshi N.D., Svinkina T., Mordes D.A., Mok J., Charlton M. (2018). The C9orf72-interacting protein Smcr8 is a negative regulator of autoimmunity and lysosomal exocytosis. Genes Dev..

[112] Ugolino J., Ji Y.J., Conchina K., Chu J., Nirujogi R.S., Pandey A., Brady N.R., Hamacher-Brady A., Wang J. (2016). Loss of C9orf72 Enhances Autophagic Activity via Deregulated mTOR and TFEB Signaling. PLoS Genet..

[113] Sharma A., Varghese A.M., Vijaylakshmi K., Sumitha R., Prasanna V.K., Shruthi S., Chandrasekhar Sagar B.K., Datta K.K., Gowda H., Nalini A. (2016). Cerebrospinal Fluid from Sporadic Amyotrophic Lateral Sclerosis Patients Induces Mitochondrial and Lysosomal Dysfunction. Neurochem. Res..

[114] Vollrath J.T., Sechi A., Dreser A., Katona I., Wiemuth D., Vervoorts J., Dohmen M., Chandrasekar A., Prause J., Brauers E. (2014). Loss of function of the ALS protein SigR1 leads to ER pathology associated with defective autophagy and lipid raft disturbances. Cell Death Dis..

[115] Al-Saif A., Al-Mohanna F., Bohlega S. (2011). A mutation in sigma-1 receptor causes juvenile amyotrophic lateral sclerosis. Ann. Neurol..

[116] Petrozziello T., Tedeschi V., Esposito A., Secondo A., Dudley J., Berliocchi L. (2017). Pharmacology of Amyotrophic Lateral Sclerosis: Old Strategies and New Perspectives. Drug Repositioning: Approaches and Applications for Neurotherapeutics.

[117] Rusmini P., Cortese K., Crippa V., Cristofani R., Cicardi M.E., Ferrari V., Vezzoli G., Tedesco B., Meroni M., Messi E. (2019). Trehalose induces autophagy via lysosomal-mediated TFEB activation in models of motoneuron degeneration. Autophagy.

